# Chordoma: A Comprehensive Systematic Review of Clinical Trials

**DOI:** 10.3390/cancers15245800

**Published:** 2023-12-11

**Authors:** Sonja Chen, Ruben Ulloa, Justin Soffer, Roberto J. Alcazar-Felix, Carl H. Snyderman, Paul A. Gardner, Vijay A. Patel, Sean P. Polster

**Affiliations:** 1 Department of Neurosurgery, University of Chicago, Chicago, IL 60637, USA; sonjac@uchicago.edu (S.C.); ralcazarf@uchicago.edu (R.J.A.-F.); 2 Department of Otolaryngology—Head and Neck Surgery, Washington University in St. Louis, St. Louis, MO 63110, USA; ulloa@wustl.edu; 3 Department of Otolaryngology—Head and Neck Surgery, University of Tennessee Health Science Center, Memphis, TN 38104, USA; jsoffer@UTHSC.edu; 4 Department of Otolaryngology—Head and Neck Surgery, University of Pittsburgh Medical Center, Pittsburgh, PA 15219, USA; snydermanch@upmc.edu; 5 Center for Cranial Base Surgery, University of Pittsburgh Medical Center, Pittsburgh, PA 15219, USA; gardpa@upmc.edu; 6 Department of Otolaryngology—Head and Neck Surgery, University of California San Diego, La Jolla, CA 92093, USA; vpatel2@rchsd.org; 7 Division of Pediatric Otolaryngology, Rady Children’s Hospital—San Diego, San Diego, CA 92123, USA

**Keywords:** chordoma, clinical trials, endonasal, endoscopic, notochord tumor, skull base, systematic review

## Abstract

**Simple Summary:**

Surgery is the primary treatment for chordoma. However, residual or recurrent chordoma presents a significant problem for clinicians. The aim of this work is to highlight the current treatment landscape for chordoma, with a particular focus on active and completed clinical trials. Future research efforts will need to address knowledge gaps including natural history studies on this disease.

**Abstract:**

This systematic review aims to characterize ongoing clinical trials and therapeutic treatment options for chordoma, a rare notochordal remnant tumor that primarily affects the cranial base, mobile spine, and sacrum. While radical surgical resection remains the cornerstone for chordoma management, unique technical challenges posed by its proximity to critical neurovascular structures confer a tendency towards disease recurrence which often requires additional treatment modalities. In an attempt to better understand the current treatment landscape, a systematic review was designed to identify clinical trials directed at chordoma. A total of 108 chordoma trials were identified from four clinical trial databases; fifty-one trials were included in the final analysis, of which only 14 were designated as completed (27.5%). Aggregate data suggests most chordoma interventions are repurposed from other neoplasms that share common molecular pathways, with a recent emphasis on combination therapeutics within and across drug classes. Naturally, the publication and dissemination of clinical trial results remain a concern (*n* = 4, 28.6%), highlighting the need for enhanced reporting and transparency measures. Active clinical trial efforts are quite promising, with a renewed focus on novel biotherapeutic targets and deciphering the natural history, as well as survivorship of this complex disease.

## 1. Introduction

**Figure 1:**
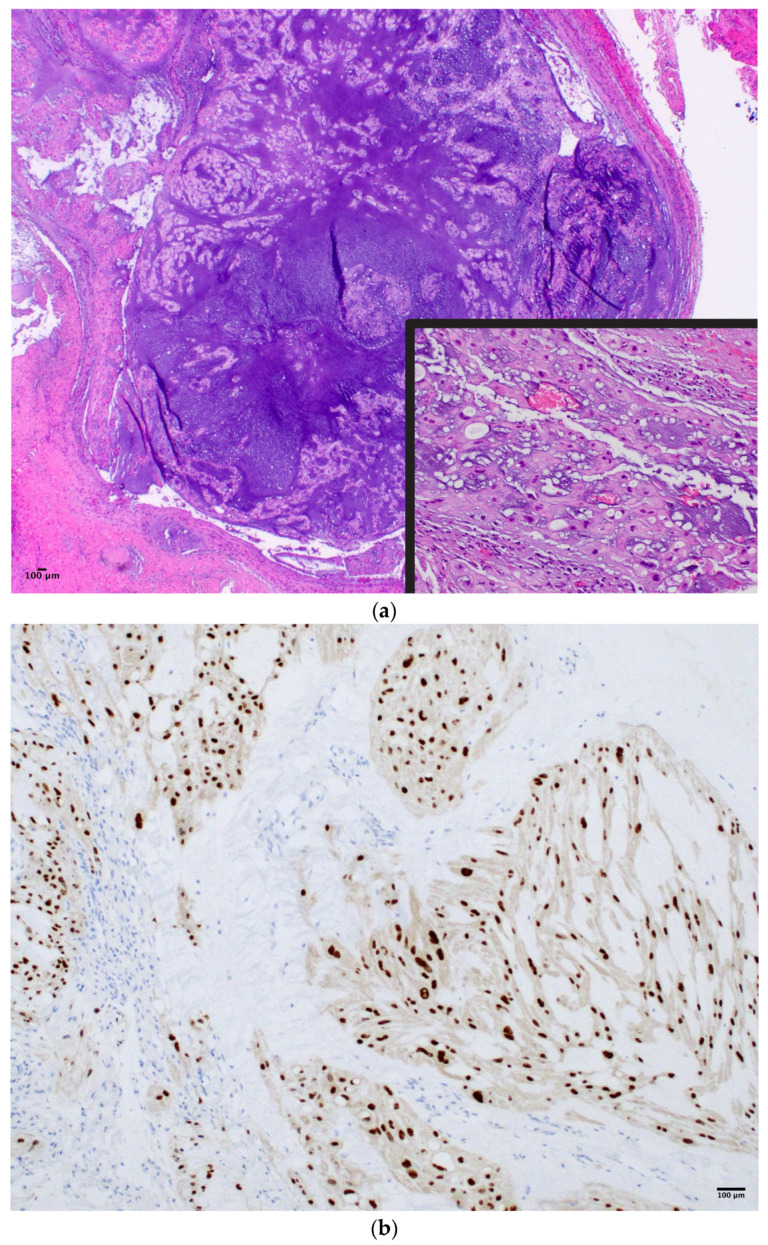
(**a**) Irregular epithelial cell clusters and interlacing strands of vacuolated physaliphorous cells in a mucoid matrix in a case of clival chordoma (H&E 4×, 20×). (**b**) Immunopositivity for Brachyury in chordoma (IHC, 20×).

Chordoma (CH), a notochordal remnant tumor of the neuroaxis, has a reported incidence of 0.3–0.8 per million persons per year worldwide [1,2,3,4]. Conventional CH is a slow-growing, indolent neoplasm. The potential for nodal and distant metastasis arises more frequently for aggressive CH subtypes with dedifferentiated histologic features [5]. Pathologically, CH cells are often epithelial and physaliphorous in appearance (Figure 1a) [6]. Disease distribution across anatomic sites includes the cranial base (26–32%), the mobile spine (23–32.8%), and the sacrum (45–29.2%) [2,4].

**Figure 2:**
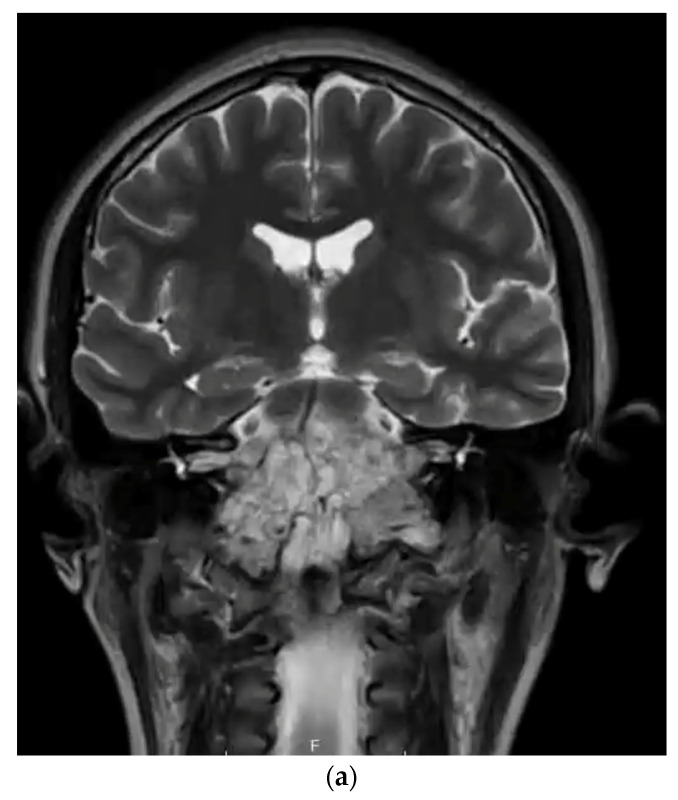

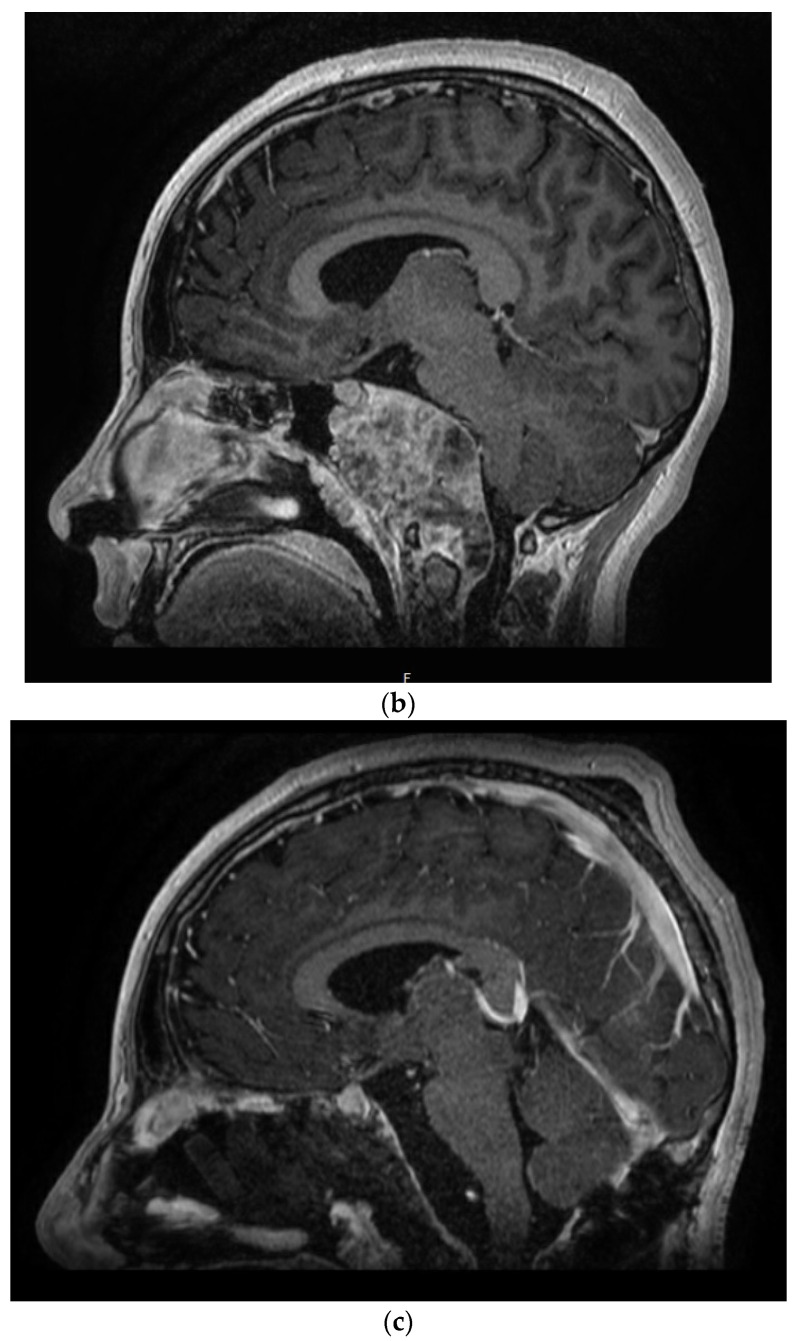
(**a**) T_2_-weighted MRI coronal sequence demonstrating the characteristic hallmarks of a clival chordoma, T_2_ hyperintensity within the skull base lesion. (**b**) T_1_-weighted MRI post-contrast sagittal sequence demonstrating anatomical boundaries of a clival chordoma with ventral brainstem compression. (**c**) T_1_-weighted MRI post-contrast sagittal sequence demonstrating gross total resection via endoscopic endonasal approach of clival chordoma.

Generally, CH symptoms vary according to its primary anatomic site. Skull base CH often presents with headaches, cranial neuropathies, and vision problems given its central involvement within the clivus, but it is often identified incidentally [7]. Sacral CH often presents with back pain, lower extremity symptoms, and/or bowel/bladder dysfunction from local compression [8]. Radical surgical resection remains the mainstay of modern treatment paradigms for CH [9] with neo-/adjuvant therapies and radiation playing a critical role in disease control [10]. Specifically, skull base CH makes complete excision particularly challenging given its proximity to critical neurovascular structures (Figure 2a,b) [11].

Interestingly, CH disease rarity has led to variable treatment patterns worldwide, which may include surgery, radiation, and/or chemotherapy. Although open ‘transcranial’ surgery is the traditionally employed modality, endoscopic endonasal approaches have been rapidly adopted in recent years given their ability to maximize the gross total resection with comparable surgical risks and outcomes (Figure 2c). Surgical advances have been important considering CH’s high recurrence rate (>50%) [12,13,14] and historically low probability of disease-free survival [15]. Unfortunately, the treatment response rate for CH adjuvant therapies is exceedingly low (0–9%) [16] which necessitates the exploration of alternative therapies.

## 2. Objective

The primary objective of this work is to create a comprehensive, up-to-date resource for clinicians by consolidating ongoing clinical trials data regarding CH adjuvant therapies. 

## 3. Materials & Methods

**Figure 3:**
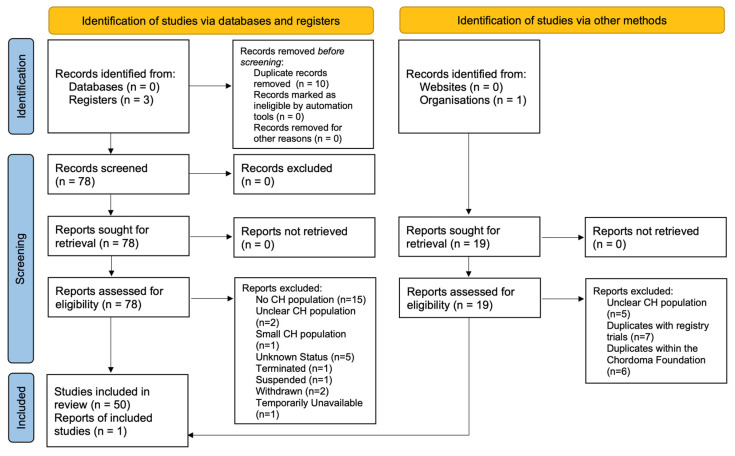
PRISMA flowchart of the inclusion and exclusion of chordoma clinical trials (1999–2022).

The National Institutes of Health’s (NIH’s) platform Clinicaltrials.gov was queried for all CH-related trials. To increase the scope of this targeted search, clinical trials listed on the Chordoma Foundation, Australian New Zealand Clinical Trials Registry (ANZCTR), and the European Union Clinical Trials Register were also cross-referenced and supplemented. This search was conducted on 30 January 2023 for all four databases. The systematic review was performed using the Preferred Reporting Items for Systematic Reviews and Meta-Analyses (PRISMA) Protocol (Figure 3) [17]. The key term “chordoma” was utilized to identify all CH-subsite-related clinical trials (*n* = 108). Exclusion criteria includes duplicate studies (*n* = 23), trials not including CH as a condition (*n* = 15), temporarily unavailable/unknown status/withdrawn/termination/suspension (*n* = 11), lack of clarity concerning the participation of CH patients’ post-trial completion (*n* = 7), and a small CH population (*n* = 1). The trials were reviewed by all authors independently to determine the eligibility for inclusion, exclusion, and risk of bias.

**Figure 4:**
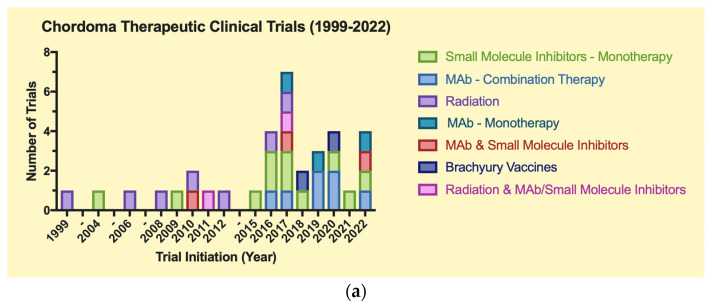

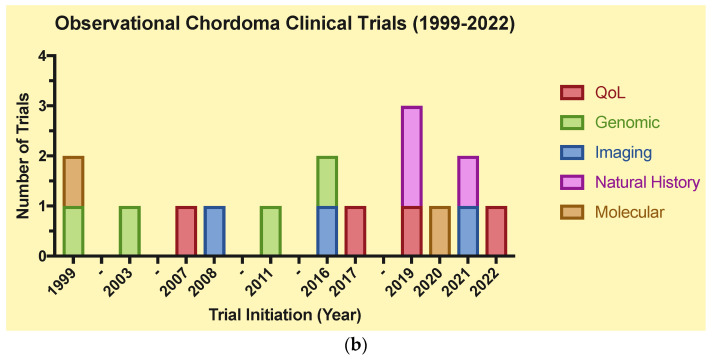
(**a**)Therapeutic clinical trials for chordoma (1999–2022). (**b**) Observational clinical trials for chordoma (1999–2022).

The clinical trials were grouped in Figure 4 by trial type: therapeutic or observational. Drugs and biologics were split into small molecule inhibitors and monoclonal antibodies. As for observational trials, “genomic” consists of gene mapping studies, while “molecular” consists of immunohistochemical and biomarker analysis studies.

## 4. Results

### 4.1. Clinical Trials Data

A total of 51 trials were included for the final analysis, which were split into two main groups: therapeutic (*n* = 35) and observational (*n* = 16) (Table 1a,b). Of these trials, 14 were completed (27.5%). The distribution of CH trials over time demonstrates a trend towards an increased number of CH-related trials between 2016–2022 (*n* = 34, 68%) vs. 1999–2004 (*n* = 5, 10%) and 2006–2015 (*n* = 11, 22%). Additionally, there is an increasing frequency of observational clinical trials for CH from 2019–2022 vs. 1999–2018. Certain time points (2018, 2021) exhibit a scarcity of CH trials, as certain intervals without notable trial progress spanned from 1999–2003, 2004–2006, and 2012–2015 (Figure 4).

### 4.2. Therapeutic Clinical Trials (Table 1a)

#### 4.2.1. Biologics & Drugs

##### Monoclonal Antibody (mAb)-Based Immunotherapy

Tumor cells may contain unique or overexpressed antigens that mAbs target, resulting in cell death and anti (α)-tumor immune responses, aiding in disease control. The safety and efficacy of two mAbs, α-PD-1 and α-EGFR, have been explored in CH. Nivolumab (α-PD-1) has since been repurposed to manage treatment-resistant CH, as 62% of CH specimens have been shown to express a high molecular weight-melanoma associated antigen [69]. One CH-related trial assessed the maximum tolerated dosage of Nivolumab (induction & q3wk) and ABI-009 (mTOR inhibitor, q2-3wk) [20]. The efficacy of Nivolumab as monotherapy or combined with Ipilimumab (α-CTLA-4 mAb) [21,22] or Relatlimab (α-LAG-3 mAb) [23] is also being assessed. Nivolumab is also being assessed in combination with immunotherapies [24] and specifically being evaluated as radiosensitizers in combination with SRS [25]. Currently, Pembrolizumab, FAZ053, PDR001, and Atezolizumab (α-PD-1 mAbs) are also being assessed as a potential for CH treatment as either monotherapy or combination therapy [18,19,26].

Cetuximab induces tumor cell apoptosis by blocking ligand binding and receptor dimerization and has previously gained FDA approval for recurrent or advanced head and neck tumors [70]. Cetuximab (α-EGFR, q1wk) is being assessed for safety and efficacy in advanced CH [27]. It is also being tested as a possible CH treatment as EGFR signaling is present in the majority of CH cases (81%) [71], with four of the seven CH cell lines being sensitive to EGFR inhibition [72]. Of note, this trial [27] is not using EGFR positivity as an inclusion criterion, given uncertainty surrounding the necessity and/or sensitivity of this particular biomarker. As immunotherapy gains popularity in other cancers, future studies directed at its effect on CH are urgently needed.

##### Enhancer of Zeste Homolog 2 (EZH2) Inhibitors

Tazemetostat, initially FDA-approved for epithelioid sarcoma, is an epigenetic regulator and an EZH2 inhibitor that is expressed in various neoplasms [73]. Its upregulation is found to cause the loss of SMARCB1/INI1, which is a key driver in pediatric CH [74]. An actively recruiting study is assessing the safety and tolerability of Tazemetostat monotherapy (BID) and in combination with mAbs (Ipilimumab, Nivolumab) in SMARCA3- or INI1-deficient CH [30]. Additionally, the efficacy of Tazemetostat (800 mg/m^2^, BID) is being evaluated in INI1-negative [28] or EZH2 gain of function mutations [29]. Early results for this INI1-negative trial suggest Tazemetostat harbors promising α-tumor activity; poorly differentiated CH patients (*n* = 6) enrolled in the dose escalation part of this trial had an objective response rate of 33% [75].

##### Tyrosine Kinase Inhibitors (TKIs)

TKIs regulate cell growth, differentiation, metabolism, and apoptosis with multiple clinical applications across various neoplasms. Meng et al. conducted a systematic review of targeted therapies in CH and concluded that TKI monotherapy should be considered a first-line treatment for CH [76]. EGFR and PDGFRβ, targets of TKIs, serve as negative prognostic factors for CH, hence the excitement around future studies aimed at assessing drug therapy in aggressive CH [77,78]. Imatinib (800 mg/day) targets PDGFRβ [76] and BCR-ABL1 fusion genes [79] and has been evaluated for efficacy towards CH [35]. A completed phase II study in 2008 (*n* = 50) confirmed its α-tumor activity in PDGF and/or PDGFβ-positive CH; 35 patients (70%) had stable disease with a 64% clinical benefit rate (i.e., partial/complete response or stable disease) and a median progression-free survival of 9 months [80]. Another ongoing study explored the safety and efficacy of Imatinib in combination with RAD001 (cell cycle inhibitor) for CH [36]. The efficacy of other TKIs that inhibit BCR-ABL (Nilotinib, 200–400 mg BID) [31] and EGFR/HER2 (Lapatinib, 410 mg [32]; Afatinib 40 mg/day for 4 weeks [33,34]) are also being investigated as monotherapies [32,33,34] or in combination with radiation therapy [31], for advanced or EGFR/HER-Neu positive CH. Finally, the safety and efficacy of Sorafenib (800 mg/day) and Regorafenib (160 mg/day, 21/28-day cycle) were evaluated for advanced and meta-static CH [37]. Sorafenib had a 73% 9-month progression-free rate [81] and Regorafenib had a 40% (*n* = 6/14) 6-month progression-free survival (no benefit concluded) [82]. Long-term studies are necessary to assess the clinical benefit of TKIs and are an active area of on-going CH research. 

##### Cyclin-Dependent Kinases (CDK) Inhibitors

CH cell lines and tissue specimens consistently demonstrate p16 (tumor suppressor protein) deletions with subsequent abnormal CDK4/6/9 activity [83,84,85,86]. Palbociclib, a CDK4/6 inhibitor, was trialed for low-to-normal p16-expressing CH to determine progression-free survival (125 mg/day for 21/28-day cycle) [40] and efficacy via disease control rate (120 mg/day for 21/28-day cycle) [39]. The CDK9 inhibitor, KB-0742, also disrupts brachyury expression in CH and was assessed for safety and tolerability in patients with relapsed/refractory solid tumors [38]. CH patients are actively being recruited in all trials.

##### Brachyury-Targeting Interventions (Figure 5)

Brachyury, uniquely expressed in CH, serves as an extremely useful diagnostic biomarker (Figure 1b, see Section 4.3.2) [87]. BN-Brachyury, a vector-based vaccine, was investigated synergistically with radiation therapy to determine if a cytotoxic T-lymphocyte-mediated immune response would improve treatment outcomes for CH. The clinical trial data revealed 10 out of 29 patients completed the trial with an objective response of 7.75% [42]. Another brachyury vaccine, TAEK-VAC-HerBy, is actively recruiting to assess the frequency of dose-limiting toxicities. It is administered in two stages for patients with brachyury and/or HER2 tumors [41]. 

**Figure 5:**
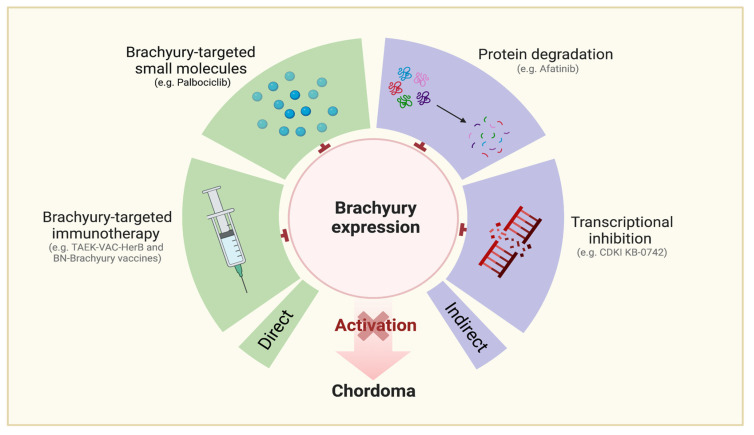
Molecular targets related to chordoma.

##### Ancillary Agents

α-folate inhibitor Pemetrexed reduces the thymidine available for DNA synthesis. Pemetrexed (900 mg/m^2^, q3wk) was assessed [44] with preliminary data, indicating a promising safety and tolerability profile in CH (*n* = 15) [88]. Similar parameters of safety and tolerability were also assessed for the cell-penetrating enhancing agent INT230-6 as monotherapy (phase I) and in combination with α-PD-1 and α-CTLA-4 mAbs (phase II) for CH. 

Preclinical studies have demonstrated INT230-6’s efficacy against large tumors and its ability to kill injected tumors and elicit a local T-cell-mediated immune response in animal models [43]. Completed in 2023, an abstract for a human phase I trial affirms INT230-6’s rapid tumor killing and immune activation properties [89]. Safety and tolerability are also being assessed for ERAS-601, an oral allosteric SHP2 inhibitor, as a monotherapy or in combination with Cetuximab and Pembrolizumab [45]. These novel agents are actively under study and may provide benefit to the CH armamentarium.

#### 4.2.2. Radiation (Table 1a)

Radiation has always been an important modality in the treatment of CH at primary and distant metastatic sites. To review radiation treatments with active clinical trial data, we divided the trials by modality: photon beam radiation therapy (*n* = 1), stereotactic body radiation therapy (SBRT) (*n* = 1), intensity-modulated radiotherapy (IMRT) (*n* = 1), and charged particle radiotherapy (*n* = 3).

Photon beam radiation therapy is the most commonly employed radiation modality; the high-energy particles damage cancer cell DNA [90]. A clinical trial assessed the combined safety and efficacy of photon beam and proton beam therapy in skull base CH [46]. 

SBRT works by a similar mechanism to subsites of interest with a completed trial by delivering a high dose of radiation [47]. 

IMRT manipulates particle beams to conform to the tumor shape and reduce collateral damage to healthy tissue [91]. An active trial for CH aims to determine the efficacy of IMRT alone or in combination with surgery via local control [48]. 

Charged particle radiotherapy trials compare multiple modalities (carbon vs. photon or carbon vs. photon & proton therapies) [50]. Another active CH trial evaluates the feasibility of proton therapy [51]. Lastly, the acute toxicity of mostly proton ion radiation therapy was measured to determine if an increased dose would reduce the chance of tumor recurrence (completed, no results/publications) [52]. 

To date, no data supports any specific modality over another for the treatment of CH. 

### 4.3. Observational (Table 1b)

The observational trials were divided into two groups based on each trial’s main topic of interest: genomic/molecular (*n* = 6) and quality of life/national history (*n* = 4).

#### 4.3.1. Imaging

Previous studies suggest the radioresistance of hypoxic tumors [92,93] which motivated two trials using hypoxia-PET and radioactive substance FMISO (completed, no results/publications) [53] and CT/MRI and PET with tracer [18F]FAZA (active) [54]. Only preclinical data is available for the [18F]FAZA trial [94]. These two trials aim to visualize hypoxic tumor areas to mitigate radioresistance and improve local control. A third imaging trial observes whether MRI parameters change within 6 months after starting proton beam therapy and before volumetric changes (recruiting) [55]. 

#### 4.3.2. Genomic/Molecular

Brachyury has served as an important diagnostic biomarker for CH; however, there is a knowledge gap surrounding the genetic drivers for CH [95]. Immunohistochemical [60] and biomarker analysis [61] clinical trials are being conducted to better understand CH protein expression. Additionally, gene mapping studies that localize inheritable diseases on chromosomes aim to test CH families for inherited autosomal dominant gene mutation [56] and map a previously identified familial CH gene to chromosome 7q933 [57]. Lastly, two trials assess the frequency of alterations in susceptibility genes [58] and the frequency of individual mutated genes [59] in CH.

#### 4.3.3. Quality of Life (QoL)/Natural History

In 2018, the NCI initiated a natural history and biospecimen collection study for rare tumors to define disease course and survival patterns [67]. Preliminary data from a pediatric/young adult CH clinic (*n* = 12, <40 years) confirmed higher success rates in localized pediatric conventional CH cases when treated with upfront resection and radiation, consistent with adult treatment recommendations. Additionally, the study identified differences in systemic therapy responses and imaging presentations between poorly differentiated and conventional CH [96]. 

A second NCI-funded natural history was initiated in 2019, which aims to collect clinical presentation, patterns of disease recurrence/progression, and response to therapies, including QoL measures, for skull base and spinal CH (recruiting, goal enrollment 300) [66].

A prospective longitudinal QoL study aims to assess patient changes following endoscopic skull base surgeries [62]. Another prospective QoL study is following pelvic bone neoplasms undergoing carbon ion radiation therapy [63]. Both trials utilize PROMIS-19 QoL measures and are currently recruiting CH patients. 

With the advent of proton therapy becoming a popular CH treatment modality, three observational radiation trials are also assessing QoL parameters. Specifically, data collected includes the efficacy and side effects of proton beam therapy (completed, no results/publications) [64], relapse-free survival of sacral CH post radiation (charged particle radiotherapies, photon & proton therapy) and/or surgical treatment (recruiting) [65], and long-term toxicities of photon and proton therapy (recruiting) [68]; QoL tools employed include chart review [64,68] and FACT-G & BIP [65]. 

## 5. Discussion

Despite advances in various adjuvant therapies for CH, there is significant fragmentation in understanding the current treatment landscape for CH patients. As with other rare diseases, variability in surgical philosophy, radiation capabilities, and medical therapies for CH has demonstrated significant treatment heterogeneity worldwide. Unfortunately, there is a dearth of completed clinical trial data to propel modern treatment decisions in CH. Specifically, among the completed clinical trials (*n* = 14, 27.5%), only four had published results (28.6%). Given these findings, it has been challenging to create a consensus statement/clinical guidelines regarding adjuvant therapies for CH. 

Historically, the majority of therapeutic agents for CH were primarily developed for other malignancies and repurposed due to the recognition of shared molecular pathways. This study indicates a move towards the predominant utilization of biologics and small molecules, with a notable prevalence of phase II clinical trials. Of all active/completed clinical trials, it appears that TKIs and EZH2 inhibitors are the most promising biotherapeutics. The efficacy of Imatinib, a TKI, demonstrated stable disease with a 64% clinical benefit rate and a median progression-free survival of 9 months [47]. Similarly, Sorafenib and Regorafenib had a 73% 9-month progression-free rate [58] and a 40% (*n* = 6/14) 6-month progression-free survival, respectively [59]. In parallel, Tazemetostat for INI-1 negative, poorly differentiated CH revealed an objective response rate of 33% [75]. Additional research efforts are necessary to further expand upon these initial short-term results.

Natural history studies are important in rare diseases given the absence of extensive databases detailing clinical symptoms, treatment outcomes, and disease progression for CH. Undertaking these studies would aid in improving the understanding of disease pathogenesis, trajectory, and evolution in CH. An interim publication of the NCI’s natural history study (NCT03739827) showcased the success of a pediatric/young adult CH clinic (*n* = 12) by collecting comprehensive data on clinical manifestations, pathology, molecular markers, and treatment interventions. It underscores the positive impact of natural history studies in connecting patients with expert physicians, offering multifaceted treatment recommendations and promoting international collaboration among experts [96].

Given the overall complexity of CH, future endeavors will likely be tied to the tumor microenvironment; the coordination of bio-banking across research institutions worldwide would enhance the accumulation of crucial tissue specimens and facilitate in-depth molecular analyses for CH. Finally, it is crucial to maximize the resource utilization amassed by the Chordoma Foundation [97]. This organization’s mission in advancing CH research and patient resources can serve as a guide for other rare conditions. 

## 6. Limitations

This systematic review exclusively addresses adjuvant therapies for CH. The rarity of CH poses challenges to clinical trials, as achieving sufficient enrolment is difficult. Consequently, CH is frequently grouped with other disease conditions in clinical trials. This constrains the conduction of rigorous analyses to develop consensus guidelines and comprehensive management principles. Ultimately, the feasibility of conducting a meta-analysis was precluded by the heterogeneity of the collected data and the absence of published reports.

A noteworthy challenge in the field is the absence of a primary classification system for CH. Various publications have grouped patients into distinct categories, such as conventional, poorly differentiated, de-differentiated [96] or low-, intermediate-, and high-grade or -risk CH groups [98]. Clinical trials have yet to differentiate among CH subtypes, although some recent trials have initiated a targeted approach based on specific molecular characteristics, such as the absence of SMARCB1 or INI expression in CHs. As an illustration, recent groundbreaking research emphasized the molecular aspects of CH and stratified patients into subgroups by using distinctions in DNA methylation patterns and whole-transcriptome expression profiles: two CH groups were identified, one being hypomethylated while the other was hypermethylated with respect to CpG islands [99]. Although still in its nascent stages, exploring variations in the tumor microenvironment and molecular biomarkers holds promise as a valuable avenue for gaining a deeper understanding of CH, warranting further investigation in future research endeavors.

Disparities in gene expression patterns and anatomical subsites may engender varying therapeutic strategies and outcomes, necessitating further investigation in forthcoming clinical trials. These distinctions may exert a substantial influence on the survival rates of advanced CH, which is not amenable to surgical intervention and is heavily reliant on adjuvant therapies. Therefore, it is reasonable for forthcoming clinical trials to stratify CH patients into distinct subgroups when presenting trial findings. The engagement of rare disease networks holds promise in catalyzing this undertaking.

## 7. Conclusions

The treatment landscape for CH currently faces fragmentation due to challenges marked by significant variability in surgical approaches, radiation capabilities, and medical therapies. Despite a lack of published data, clinical trials have demonstrated most CH therapies to be repurposed from other neoplasms that share common molecular pathways, with an increased focus on small molecules (TKIs, EZH2 inhibitors) and combination therapeutics. A recent upward trend is evident for observational trials as well. Emerging concepts in CH include risk stratification, tumor microenvironment, and biomarker discovery. To further advance our understanding of CH disease manifestations, treatment, and survival, it is imperative to engage in natural history studies and establish a comprehensive nationwide tumor registry. Collaborative research efforts between academia, pharmaceutical companies, and advocacy organizations are necessary to improve patient outcomes and develop standardized treatment guidelines [16].

## Figures and Tables

**Table 1:** (**a**) Summary of therapeutic clinical trials for chordoma. (**b**) Summary of observational clinical trials for chordoma.

(a)			
Intervention	Phase	Subcategory	Primary Outcome Measures
**Biologic + Drug**	Phase I Phase I + II Phase II	α-PD-1 mAb: FAZ053 [18] Atezolizumab [19] Nivolumab [20,21,22,23,24,25] Pembrolizumab [26]	Assess whether α-tumor activity increases when combined with PDR001 (α-PD-1 mAb) Assess efficacy when combined with Tiragolumab (α-TIGIT mAb) Determine PFS, ORR, MTD, DLT, and safety when administered in combination with other drug and radiation therapies Evaluate treatment response and ORR for unresectable metastatic CH
	Phase II	α-EGFR mAb: Cetuximab [27]	Assess efficacy by measuring ORR
	Phase I Phase II	EZH2 inhibitors: Tazemetostat [28,29,30]	Assess safety and tolerability with monotherapy vs. combination with Nivolumab & Ipilimumab Evaluate efficacy in EZH2 GOF mutation and INI1-negative tumors
	Phase I Phase II	TKIs: Nilotinib [31] Lapatinib [32] Afatinib [33,34] Imatinib [35,36] Sorafenib, Regorafenib [37]	Assess synergistic effect of concurrent RT via measuring DLT and above MTD Assess efficacy (EGFR-expressing and HER2Neu+ advanced CH via ORR) Assess efficacy (EGFR- and/or HER2- expressing CH via PFS and ORR) Assess efficacy (in combination with RAD001 (cell cycle inhibitor) on PDGFRβ+ CH Assess α-tumor activity and its effect on PDGFRβ+ CH Assess safety and efficacy for CH patients with genomic variant of the drug’s target
	Phase I Phase II	CDK inhibitors: KB-0742 [38] Palbociclib [39,40]	Assess safety and tolerability of relapsed or refractory CH via AE, MTD, and RP2D Assess efficacy (LA or metastatic CH via DCR and PFS)
	Phase I Phase II	Brachyury vaccines: TAEK-VAC-HerB [41] BN-Brachyury [42]	Assess DLT & vaccine interaction with therapeutic HER2 antibodies Assess vaccine radiographic ORR in CH in combination with radiation therapy
	Phase I Phase I + II	Other: INT230-6 [43] Pemetrexed [44] ERAS-601 [45]	Assess safety and tolerability of drug and when combined with α-PD-1 and α-CTLA-4 mAbs Assess safety and tolerability via PFS and radiographic response Assess safety and tolerability of drug and when combined with α-PD-1 and α-EGFR mAbs
Radiation	Phase II	Photon Beam Radiation Therapy [46]	Assess safety, efficacy, and LC in combination with or without PBT
	Phase IV N/A	Stereotactic Radiation Therapy [47]	Evaluate safety, efficacy, and LC
	N/A	Intensity Modulated Radiotherapy [48,49]	Assess efficacy of monotherapy vs. combination with surgery Assess ability in reducing late normal tissue toxicity and optimal radiation dose for CH
	Phase I + II Phase III N/A	Charged Particle Radiation Therapy [50,51,52]	Assess PFS of CIRT vs. proton RT or photon RT or proton & photon RT Assess the possibility of delivering an optimal dose within normal tissue tolerances Assess proton therapy’s feasibility & acute toxicity for CH patients
**(b)**			
**Intervention**	**Phase**	**Subcategory**	**Primary Outcome Measures**
**Imaging**	N/A Phase II	CT/MRI and FMISO-PET [53] CT/MRI and [18F]FAZA-PET [54]	Visualize hypoxic tumor areas via tracer to improve radiation dosing/planning
	N/A	MRI post-PBT [55]	Determine if MRI parameters change within 6 months after the start of PBT and prior to volumetric changes
**Genomic/Molecular**	N/A	Gene Mapping [56,57,58,59]	Study germline and somatic mutations associated with CH
	N/A	Immunohistochemical [60]	Retrospectively explore protein expression, including brachyury, in CH
	N/A	Biomarker analysis [61]	Biobanking network of tissue specimens
**QoL/Natural History**	N/A	Quality of Life [62,63,64,65]	Assess patient changes in QoL post-EEA skull base surgeries (PROMIS-29) Evaluate whether CIRT improves QoL post-pelvic treatment (PROMIS-29) Evaluate relapse free survival in CH patients undergoing surgery vs. RT (FACT-G, BIP) Evaluate outcome data on tumor control for cancers post-PBT
	N/A	Characterize history of skull base/spine CH [66,67]	Characterize clinical presentation and disease progression patterns
	N/A	Characterize patterns of care and long term toxicities [68]	Assess long-term effects of photon and PBT in CH patients (chart review)

Abbreviations for both Table 1a,b: AE—adverse events; BIP—Brief Inventory Pain; CDK—cyclin-dependent kinase; CIRT—carbon ion radiation therapy; CH—chordoma; CT/MRI—computed tomography/magnetic resonance imaging; DCR—disease control rate; DLT—dose limiting toxicities; EEA—endoscopic endonasal approach; EGFR—epidermal growth factor receptor; EZH2—enhancer of zeste homolog 2; FACT-G—Functional Assessment of Cancer Therapy General; FMISO—Fluoromisonidazole; GOF—gain of function; HER—human epidermal growth factor receptor; LA—locally advanced; LC—local control; mAb—monoclonal antibodies; MTD—maximum tolerable dose; Neu—neuroglioblastoma cell line; ORR—objective response rate; PD-1—programmed cell death protein 1; PBT—proton beam therapy; PFS—progression free survival; PET—positron emission tomography; PROMIS-29—patient-reported outcomes measurement information system comprising 29 items; QoL—quality of life; RP2D—recommended phase 2 dose; RT—radiation therapy; TKI—tyrosine kinase inhibitor; [18F]FAZA—[18Fluor] Fluoroazomycin Arabinofuranoside.

## Data Availability

The data can be shared up on request.

## Abbreviations

BIP—Brief Inventory Pain; CTLA-4—cytotoxic T-lymphocyte-associated protein 4; CT/MRI—computed tomography/magnetic resonance imaging; EGFR—epidermal growth factor receptor; FACT-G—Functional Assessment of Cancer Therapy General; FMISO—Fluoromisonidazole; HER—human epidermal growth factor receptor; INI1—integrase interactor 1; LAG-3—lymphocyte-activation gene 3; mTOR—mammalian target of rapamycin; Neu—neuroglioblastoma cell line; PD-1—programmed cell death protein 1; PET—positron emission tomography; PDGFRβ—platelet-derived growth factor receptor-β; PROMIS-29—patient-reported outcomes measurement information system comprising 29 items; SHP2—Src homology region 2-containing protein tyrosine phosphatase 2; SMARCA4—SWI/SNF Related, Matrix Associated, Actin Dependent Regulator Of Chromatin, Subfamily A, Member 4; SMARCB1—SWI/SNF Related, Matrix Associated, Actin Dependent Regulator Of Chromatin, Subfamily B, Member 1; TIGIT-1—T cell immunoreceptor with immunoglobulin and ITIM domain 1; VEGFR—vascular endothelial growth factor receptor; [18F]FAZA—[18Fluor] Fluoroazomycin Arabinofuranoside.
